# A Pipette for High-Resolution Sampling and Delivery of pL Bio-Samples

**DOI:** 10.3390/mi16060630

**Published:** 2025-05-27

**Authors:** Ziyang Han, Pengfei Gong, Hengxiang Su, Zehang Gao, Shilun Feng, Jianlong Zhao

**Affiliations:** 1 State Key Laboratory of Transducer Technology, Shanghai Institute of Microsystem and Information Technology, Chinese Academy of Sciences, Shanghai 200050, China; 16638076886@163.com (Z.H.); g1432115534@126.com (P.G.); gaozh@shanghaitech.edu.cn (Z.G.); 2 Xiangfu Laboratory, Jiashan 314102, China; suhengxiang@126.com; 3 Shanghai Frontier Innovation Research Institute, Shanghai 201108, China

**Keywords:** sampling pipette, biological detection, microfluidic system, microdroplet, *E. coli*

## Abstract

Conventional laboratory methods for handling valuable biological samples typically use pipettes or needles, which are prone to issues such as dead volume and sample waste. Furthermore, the sampling and processing of pathogenic bacteria, such as *Escherichia coli* (*E. coli*) in environmental wastewater, require labor-intensive procedures with multiple complex steps. To overcome these limitations, we developed a pipette integrated with a microfluidic chip for bacteria sampling and delivery. This pipette can provide the negative pressure to microfluidic chips for sampling, the constant temperature unit for biological reaction, and programs for automatic control (suction, heating, liquid discharge, and cleaning) and display. The droplet chip employs a cross-channel structure to generate droplets and incorporates a designated droplet storage and detection area. Utilizing this innovative device, we have demonstrated the generation, transportation, and storage of pL droplets, as well as quantitatively detected *E. coli* samples across various concentrations. The test results have demonstrated the overall reliability and data consistency of the system. Overall, our device achieves the precise sampling of pL volumes, offering a simple yet promising solution for the sampling and delivery of bio-samples in remote settings.

## 1. Introduction

Handling bio-samples with high precision is a complex task that involves adding extremely small volumes and storing them. This process can easily lead to contamination and present significant technical challenges [1]. Currently, most biological sampling methods rely on manual pipetting, which involves transferring samples between containers and equipment. To prevent contamination and biohazard risks, these methods require stringent environmental controls, such as sealed workspaces and high-level biosafety facilities. However, three major issues remain: (1) the system cannot be completely sealed during analysis, (2) manual sample transfer introduces a risk of cross-contamination, and (3) personnel remain at risk of viral exposure. Even the most advanced droplet microfluidic systems exhibit inherent limitations [2]. These systems require manual sample loading and use pump valve assemblies to generate droplets. Moreover, the sample transfer process can introduce contaminants, potentially affecting the accuracy of detection and interfering with subsequent microfluidic analyses [3].

Manual pipettes remain the mainstream tools in small- and medium-sized laboratories, and their design principles directly affect the accuracy of operations [4]. Air displacement pipettes operate by using a spring to drive a piston to move within a sealed chamber, achieving liquid aspiration and dispensing through the pressure difference. For example, the Eppendorf Research^®^ plus series employs a double O-ring ceramic seal that can control the volume error within ±0.5%. However, this method demands a high level of skill from the operator, who must pay attention to pre-wetting to reduce adsorption on the pipette walls. Positive-displacement pipettes utilize disposable pistons that directly contact the liquid, thereby completely eliminating the risk of aerosol contamination [5]. This makes them suitable for handling volatile substances (such as chloroform), highly viscous samples (>500 cP), or those containing suspended particles. For example, the MICROMAN^®^ E features an integrated capillary channel within its piston, achieving a coefficient of variation (CV) of ±0.8% within a 500 μL range. However, this method is relatively expensive, and the need for piston replacement may introduce operational delays. Automation systems have significantly enhanced throughput and reproducibility through mechatronic design [6], and they are primarily categorized into three types: (1) multichannel pipetting workstations are suitable for large-scale experimental screening; (2) acoustic dispensing technology propels nanoliter-sized droplets to target positions without physical contact [7]. This technology boasts a cross-contamination rate of less than 0.001% and supports reverse pipetting. However, the efficiency of acoustic transfer is influenced by the physical properties of the liquid (density and surface tension), necessitating the optimization of parameters for different samples. (3) Piezoelectric dispensing leverages the converse piezoelectric effect of piezoceramics to convert electrical energy into mechanical vibrations, which drive the liquid through a micro-nozzle to form uniform droplets [8]. The main challenge lies in the susceptibility of the nozzle to clogging, which requires the use of low-viscosity buffers (<10 cP).

The sampling and delivery of nanoliter and picoliter volumes of bulk fluids is an active area of biomedical research. Currently, no research institution or company has developed the technology or devices capable of directly collecting samples as droplets within a closed environment to enable the storage and controllable reaction of field droplet information [9]. The accurate sampling of fluids with rapidly changing composition cannot be achieved using traditional techniques. In particular, conventional needles for venous, intrathecal, and related applications, including microneedle arrays, cannot accurately transport chemicals with time-varying concentrations due to Taylor dispersion. In 1953, Taylor described this effect as a “combined effect of changes in molecular diffusion and velocity across the cross-section” [10]. This process is similar to multimodal dispersion in an optical fiber, significantly disrupting the chemical information transmitted through the channel [11]. This dispersion effect severely limits the temporal precision of the signal and makes it difficult to transport complete chemical samples from remote locations.

In a microfluidic environment, a “droplet” is defined as a large volume of fluid that naturally forms a spherical shape in another immiscible phase. Specifically, the aqueous phase forms a discrete volume that nearly fills the channel. Such microdroplets can effectively address the challenges of sampling small volumes of liquid and the information destruction caused by the Taylor effect. Studies of segmented flows in microchannels predate the recent surge in microfluidic droplet research by nearly 50 years [12]. However, their application in transmitting biochemical signals has only been demonstrated in the past decade. Feng et al. developed droplet-based microfluidics with a lower sampling dead volume and transport with higher resolution [13]. The collection, mixing, reaction, and treatment of droplets offer a novel approach for real-time field detection in biological sensing.

Tetanus, typhoid fever, pneumonia, tuberculosis, and other diseases are caused by various pathogenic bacteria. Detecting these bacteria is crucial for human health and life safety. The traditional pathogen detection method includes the plate colony counting method, in which the pathogenic bacteria samples are diluted and coated on the Petri dish. After incubation, the concentration of the original sample can be obtained by calculating the number of colonies [14]. However, the whole process exhibits the following limitations: (1) time-consuming, as 24–48 h is generally needed to obtain the results; (2) having high requirements for experimental operation and experimental conditions [15]. Therefore, the rapid detection of pathogenic bacteria technology emerged, such as immunosensors, polymerase chain reaction (PCR) technology, digital PCR, LAMP technology, etc. Immunological approaches utilizing the specific binding of antigen–antibodies effectively shorten the detection time of pathogenic bacteria. However, the detection accuracy is limited, and they can only detect the antigen within 10 days of infection, and the test results can only be qualitative. PCR technology has been developed as the gold standard for nucleic acid detection, but only one pathogen can be detected at a time, and it also requires 3 to 4 h. Digital PCR technology uses the digital detection idea to greatly improve detection accuracy. However, due to the need for repeated thermal denaturation to obtain a single-stranded template and the high cost of heating components, the method remains expensive. The LAMP technique overcomes the need for repeated temperature cycling in PCR, achieving continuous rapid amplification at a constant temperature [16]. In recent years, there have been many studies on enzymatic reactions using droplets. For example, the microfluidic enrichment and detection system developed by Gong et al. has demonstrated the rapid detection of *E. coli* through a droplet enzymatic reaction [17], but the portability of the system still needs to be improved. In general, as a new platform, droplet microfluidics has been widely used in a variety of detection research. However, due to the large and off-site droplet preparation equipment, this method is difficult to adapt to various applications [18]. Therefore, there is a need for a portable hand-held sampling device that can automatically generate and store droplets and has the ability to analyze samples in a relatively fast and sensitive way.

In this study, we have developed a droplet microfluidic sampling system for the immediate sampling and manipulation of pathogenic bacteria, which can realize the functions of closed sampling in droplet form, transportation in droplet form, and incubation. The sampling system consists of a sampling chip and a droplet sampling pipette. Droplets are generated by a sampling chip and drawn into the sampling pipette for heating cultivation. We achieve the direct extraction and delivery of small volumes of rapidly changing samples in droplet form, and the aqueous phase samples are carried as droplets by immiscible oil in the hydrophobic channel so that the samples at each moment are wrapped in independent droplets and a thermostatic digital enzymatic reaction can be performed on the chip for non-destructive, high-sensitivity detection.

## 2. Materials and Methods

### 2.1. Reagent and Consumables

The *E. coli* O157:H7 strain (ATCC 43888, Cell Bank, Chinese Academy of Sciences) was cryopreserved at −80 °C in 25% (*v*/*v*) glycerol stock prior to experimentation. For bacterial resuscitation, frozen stocks were streaked onto Luria–Bertani (LB) agar plates (Huankai Microbial, Guangzhou, China) for single-colony isolation and subsequently cultured in LB broth (Huankai Microbial, Guangzhou, China) at 37 °C under aerobic conditions. Primary inocula were prepared by transferring a single isolated colony into 10 mL of LB broth followed by aerobic incubation at 37 °C with 200 rpm orbital shaking for 14 h. Bacterial cell density was quantified spectrophotometrically at 600 nm (OD600), where an optical density unit of 1.0 corresponded to approximately 2 × 10^9^ colony-forming units (CFU)/mL, as calibrated against quantitative plate counts. Serial decimal dilutions were prepared in sterile phosphate-buffered saline (PBS, pH 7.4, Solarbio, Beijing, China), with final bacterial concentrations being quantitatively validated through the standard spread plate enumeration method on LB agar.

Mineral oil (Biotechnology grade,), Triton X-100 (BioUltra grade), and fluorescein di-β-D-galactopyranoside (FDG, ≥98% purity) were obtained from Sigma-Aldrich (Kenilworth, NJ, USA). The polymer materials included polydimethylsiloxane (PDMS, Sylgard™ 184 kit, Dow Corning, Midland, MI, USA) and ABIL EM 90 (Evonik Industries AG, Essen, Germany). The photolithographic materials included SU-8 2100 photoresist (Kayaku Advanced Materials, Westborough, MA, USA). All solutions were prepared using sterile PBS as the diluent.

### 2.2. Design and Microfabrication of Droplet-Based Microfluidic Chip

The microfluidic chip channels and microchambers were designed using AutoCAD 2023 and fabricated via soft lithography using a T-shaped structure to generate droplets. The height of the flow channel is 100 μm; the length of the sample channel is 185 mm and the width is 100 μm; and the length of the oil channel is 85 mm and the width is 225 μm. The chip mold was fabricated using conventional mask-based soft lithography. The detailed fabrication procedure is as follows: a 4-inch silicon wafer was cleaned using deionized water in a plasma cleaner and dried with a nitrogen pipette. The wafer was then subjected to plasma treatment for 1 min to remove surface contaminants, and a 100 μm thick layer of photoresist was spin-coated onto the mask using a spinner. The coated substrate was placed on a hot plate at 50 °C for 1 min, followed by a gradual temperature increase of 5 °C increments to minimize cracking, until reaching 95 °C, where it was maintained for 40 min. The substrate was then allowed to cool to room temperature for 1–2 h. The subsequent steps included exposure, development, and hard baking to obtain the final chip mold.

A total of 10 g of PDMS (base/curing agent ratio of 10:1) was poured into the center of the mold and degassed in a vacuum chamber for 1 min to form a 500 μm thick film. After curing at 85 °C for 2 h, the PDMS film was carefully peeled off the mold and cut to the required dimensions [19]. The PDMS was bonded to glass via plasma treatment. Following chip bonding, the assembly was heated at 105 °C for 4 h to enhance the surface hydrophobicity of the internal PDMS channels for subsequent use [20].

### 2.3. Design and Manufacture of Sampling Pipette

The droplet sampler consists of a pipette tip module, a heated tubing module, a battery pack, a sliding cylinder, and a circuit board control module. The sampler’s pipette tip is designed to accommodate standard syringe needles of varying sizes.

The cylinder used is the PB10X40R (AirTAC, Taiwan, China), featuring a 10 mm bore diameter, a 40 mm stroke, and axial air intake. The sampler employs a Bugatti miniature stepper motor sliding stage, model 44-SM15-80L-T. This stage is driven by a 2-phase, 4-wire motor with an operating voltage of 4–9 V and a current of 100–500 mA. It includes a 90 mm lead screw with an 80 mm travel range, a 15 mm motor diameter, a 3 mm lead screw diameter, a 3 mm smooth shaft diameter, and a 0.5 mm lead screw pitch. The motor has a step angle of 18° and a phase resistance of 15.5 Ω, with overall dimensions of 105 mm × 15 mm × 18 mm. As a stepper motor, it requires a stepper motor driver and control board to operate. The display module utilizes a 1.5-inch OLED screen from Zhongjingyuan Electronic Technology Co., Ltd. (Zhengzhou, China). The heating module incorporates a PI polyimide resistive heating film from Jinxing Technology Co., Ltd. (Harbin, China), sized 20 mm × 50 mm, with a rated voltage of 12 V and a power rating of 5 W.

### 2.4. Droplet Formation and Biological Monitoring Process

**Figure 1:**
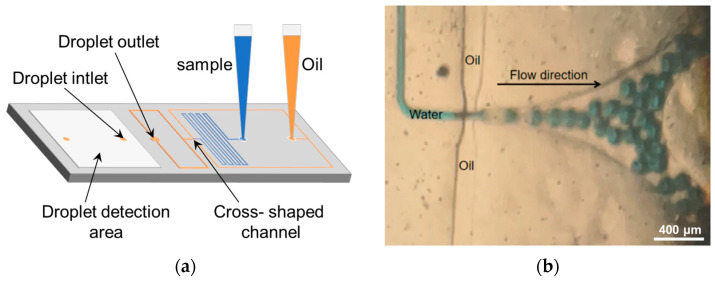
(**a**) Schematic diagram of the PDMS chip structure. (**b**) Diagram of droplet generation process. Scale bar is 400 μm.

Firstly, we used a regular pipette to extract the sample and oil separately and inserted them into the chip (Figure 1a), and then we left only the pipette tips containing the sample and oil on the slide.

The sampling pipette generates negative pressure to drive the oil and water phases within the chip toward the cross-shaped junction, where droplets are formed and bacteria in the aqueous phase are encapsulated [21]. Once generated, the droplets are stored in a capillary tube integrated within the sampling pipette. The droplets are subsequently heated by the tube heating module to facilitate enzymatic reactions. Upon the completion of the enzymatic reaction, the sampling pipette is connected to the detection zone of the droplet chip. The droplets are then distributed as a monolayer within the detection zone for subsequent fluorescence-based detection.

## 3. Results

### 3.1. Simulation of Droplet Formation

In the design of droplet-generating structures, flow field focusing structures, which are frequently utilized, necessitate the separation of the two-phase reagents. Currently, most droplet-generating structures predominantly rely on positive pressure to produce droplets [22]. However, the ultimate objective of this paper is to develop an integrated device capable of collecting samples, generating droplets, and conducting detections. Therefore, we opt to transition the droplet-generating mechanism from positive pressure to negative pressure drive, thereby facilitating the seamless integration of the device.

The challenge in negative pressure-driven droplet generation lies in regulating the flow resistance of the two-phase fluid channel to maintain an optimal flow rate for oil and water, thereby ensuring stable droplet formation. This differs from positive pressure droplet formation, which primarily adjusts the two-phase flow rates through pressure control [23]. In negative pressure systems, achieving appropriate droplet formation necessitates a higher degree of flow resistance matching in channel design. Each channel’s length is meticulously designed based on fluid resistance calculations. Fluid resistance plays a crucial role in determining flow rate matching among the sample, reagent, and oil phases, enabling the control of suitable water–oil flow rates [24]. This allows the water phase to generate droplets at appropriate intervals within the oil phase. Additionally, the droplet formation can be calculated using the following equation:

(1) $$R=12\mu \frac{L}{WH^{3}\left(1-0.63\frac{H}{W}\right)}$$

**Figure 2:**
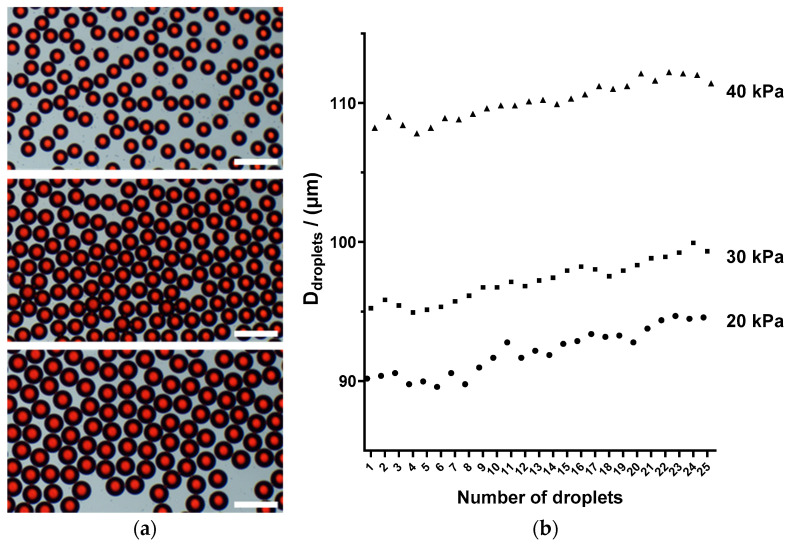
(**a**) Microscopic images of droplets generated under negative pressures of 20, 30, and 40 kPa. (**b**) Droplet size distribution picture generated under negative pressures of 20, 30, and 40 kPa. Scale bar is 250 μm.

In Equation (1), *L*, *W*, and *H* represent the length, width, and height of the microfluidic channel, respectively, while *μ* denotes the viscosity of the fluid and *R* denotes the fluidic resistance. The oil phase employed in this study is mineral oil, exhibiting a fluid viscosity of 25 mPa·S, whereas the water phase has a fluid viscosity of 1 mPa·S. Upon the completion of the microfluidic chip (Figure 1a), testing was conducted on its droplet generation within a pressure range of 5–75 kPa. The results indicated that stable droplet generation occurred at a rate of approximately 100 droplets per second near the pressure range of 20–40 kPa. The diameter statistics of 25 droplets generated under 20, 30, and 40 kPa negative pressure show good uniformity (Figure 2).

### 3.2. Assembly and Display of Sampling Pipette

In this article, the characteristics required for a qualified pL biological sample droplet sampling pipette include a compact size for easy portability; sufficient pressure generation capability to drive microfluidic chips; an internal heating module to facilitate sample heating for further analysis; and a user-friendly operating system that allows for swift and effortless setting adjustments. Ultimately, we have developed a droplet sampling pipette that integrates suction, discharge, heating, and storage functions into one device. The functional components of this sampling pipette comprise a nozzle, a conduit heating module, a battery pack, a sliding cylinder, and a circuit board control module.

**Figure 3:**
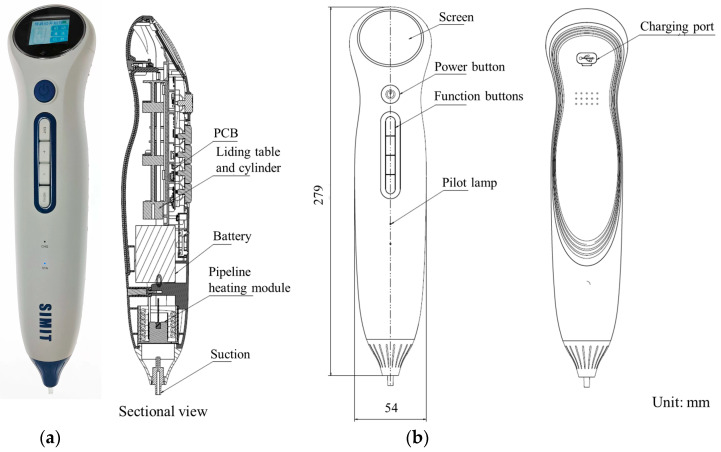
(**a**) Physical picture of the sampling pipette. (**b**) Design diagram of sampling pipette.

Finally, the product we have obtained is depicted in Figure 3. It boasts a sampling negative pressure range of 5 to 75 kPa, a suction flow rate range of 30 to 180 μL/s, a discharge flow rate range of 10 to 60 μL/s, and a temperature variation range of 30 to 50 °C. This product is capable of controlling the pressure and flow rate of liquid suction, storing the aspirated droplets within its body for constant temperature heating, and then discharging them in a controlled manner after a preset period, thereby proceeding to the next detection stage. The various specifications of this product fully meet the requirements of the sampling chip, and its parameters can be flexibly adjusted to accommodate diverse sampling needs.

### 3.3. Droplet Sampling Pipette–Chip Connection and E. coli Detection

**Figure 4:**
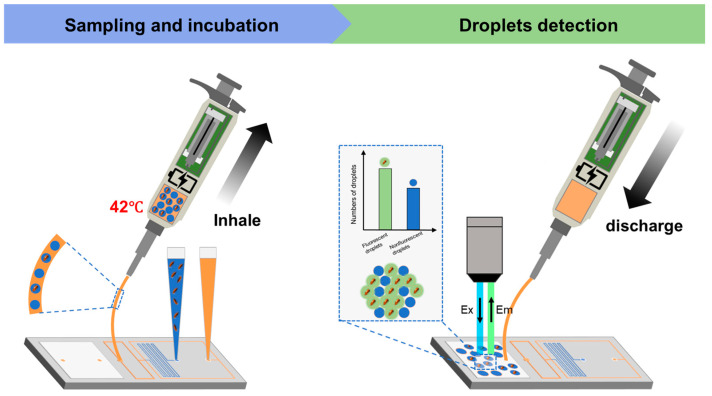
Schematic diagram of sampling pipette operation.

**Figure 5:**
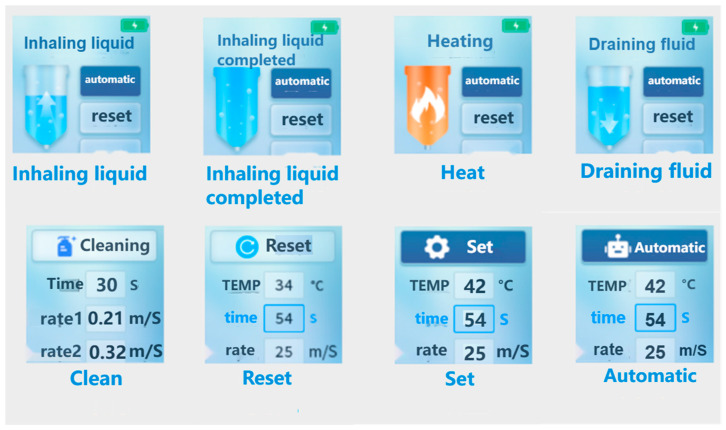
Operation interface flowchart of the sampling pipette.

We can establish a comprehensive closed-loop system for fluorescent droplet sampling and enzymatic incubation by connecting the sampling pipette and droplet chip via a hose. To assess the fidelity of the system’s fluorescence intensity testing, we developed an *E. coli* fluorescence intensity detection system. The fluorescent sample is propelled by the negative pressure of the sampling pipette, passes through the sampling chip to form droplets, and then enters the sampling pipette via the hose. After being heated at 42 °C for 4 h, the sample is discharged and directed through another hose to a storage area, where it is photographed and counted under a microscope (Figure 4). The entire process is displayed in real time on the sampling pipette’s display screen (Figure 5).

**Figure 6:**
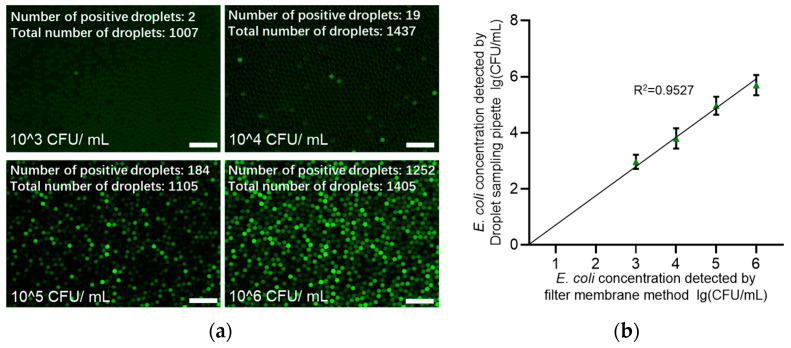
(**a**) Fluorescence images of liquid droplets and their counting results. (**b**) Linear fitting curve of fluorescence counting results. Scale bar is 250 μm.

The catalytic conversion of FDG to fluorescein by GAL, released by *E. coli* cells, induces strong fluorescence in the droplets at an excitation wavelength of 485 nm and an emission wavelength of 535 nm. Consequently, the presence of fluorescent droplets is attributed to the presence of bacteria. The distribution of each cell is random and independent, and the number of cells in each droplet is also random and independent [25]. Therefore, the number of cells in a droplet follows a Poisson distribution (Equation (2)). The viable bacterial count is determined by enumerating these positive droplets (Equation (4)). Following the sampling and incubation process using the microfluidic chip-based system (encompassing sample generation, droplet sampling, storage, and detection areas), a linear correlation was observed between the number of fluorescent droplets and the initial concentration of *E. coli* (Figure 6). This finding underscores the system’s consistency and suitability for biological sampling applications.

(2) $$P(x=k)=\frac{e^{-\lambda }}{k!}\lambda^{k}$$

(3) λ=C V

Thus,

(4) $$bacteria\;concentration=\frac{ln(1-\frac{positive}{total})}{V}$$

In Equation (2), *P* (*x* = *k*) is the probability to have *k* cells per droplet and *λ* is the mean number of cells per droplet. In Equation (3), *C* represents the concentration of bacterial cells and *V* is the average volume per pico-droplet.

## 4. Conclusions

We propose a microdroplet-based sampling and detection solution to address the challenges of detecting pL biological samples. A PDMS microfluidic chip was designed to generate uniform pL droplets by encapsulating samples in oil under negative pressure. Additionally, a portable microfluidic droplet sampling pipette was developed to provide negative pressure and stable heating, with adjustable parameters to meet diverse sampling needs.

The integrated system, comprising the microfluidic chip and droplet sampling pipette, was validated using *E. coli* for fluorescence detection. The fluorescence intensity exhibited a linear correlation with the initial bacterial concentration, confirming the system’s consistency and reliability. This innovative approach holds great promise for the sampling, processing, and detection of pL biological samples, offering a robust and versatile solution for future applications.

## Data Availability

The original contributions presented in this study are included in the article. Further inquiries can be directed to the corresponding authors.

## Abbreviations

The following abbreviations are used in this manuscript:

*E. coli*	*Escherichia coli*
pL	Picoliter
POCT	Point-of-Care Testing
FDG	Fluorescein di(β-D-galactopyranoside)
GAL	β-Galactosidase
PCR	Polymerase chain reaction
LAMP	Loop-mediated Isothermal Amplification
PDMS	Polydimethylsiloxane

## References

[1] Hansen G., Marino J., Wang Z.X., Beavis K.G., Rodrigo J., Labog K., Westblade L.F., Jin R., Love N., Ding K. (2021). Clinical Performance of the Point-of-Care cobas Liat for Detection of SARS-CoV-2 in 20 Minutes: A Multicenter Study. J. Clin. Microbiol..

[2] Sah A.K., Joshi B., Khadka D.K., Gupta B.P., Adhikari A., Singh S.K., Rai G., Vaidya G.S., Rajbhandari R., Pant B. (2017). Comparative Study of GeneXpert MTB/RIF Assay and Multiplex PCR Assay for Direct Detection of Mycobacterium tuberculosis in Suspected Pulmonary Tuberculosis Patients. Curr. Microbiol..

[3] Sun Y., Ma C., Wu M., Jia C., Feng S., Zhao J., Liang L. (2022). Sensitivity of photoelctrocehmical aptasensor using spiral nanorods for detecting antiobiotic levels in experimental and real samples. Talanta.

[4] Kusonpan P., Kunpatee K., Chailapakul O., Kalcher K., Ortner A., Chaiyo S., Samphao A. (2025). A simple manually rotated paper-based analytical device with electrochemical sensors for the determination of nitrite and nitrate. Talanta.

[5] Siri Y., Sthapit N., Malla B., Raya S., Haramoto E. (2024). Comparative performance of electronegative membrane filtration and automated concentrating pipette for detection of antibiotic resistance genes and microbial markers in river water samples. Sci. Total Environ..

[6] Blums K., Herzog J., Costa J., Quirico L., Turber J., Weuster-Botz D. (2025). Automation of RNA-Seq Sample Preparation and Miniaturized Parallel Bioreactors Enable High-Throughput Differential Gene Expression Studies. Microorganisms.

[7] Zhu H., Deng Q., Li J., Yang L., Li H., Zhao Z., Wang Z., Pang C., Zhang Y., Lui V.C. (2025). Sound-controlled fluidic processor. Sci. Adv..

[8] Mau R., Seitz H. (2023). Influence of the Volatility of Solvent on the Reproducibility of Droplet Formation in Pharmaceutical Inkjet Printing. Pharmaceutics.

[9] Jacobs C.B., Peairs M.J., Venton B.J. (2010). Review: Carbon nanotube based electrochemical sensors for biomolecules. Anal. Chim. Acta.

[10] Bontidean I., Ahlqvist J., Mulchandani A., Chen W., Bae W., Mehra R.K., Mortari A., Csöregi E. (2003). Novel synthetic phytochelatin-based capacitive biosensor for heavy metal ion detection. Biosens. Bioelectron..

[11] Datsenko K.A., Wanner B.L. (2000). One-step inactivation of chromosomal genes in *Escherichia coli* K-12 using PCR products. Proc. Natl. Acad. Sci. USA.

[12] Huang C.M., Zhu Y., Jin D.Q., Kelly R.T., Fang Q. (2017). Direct Surface and Droplet Microsampling for Electrospray Ionization Mass Spectrometry Analysis with an Integrated Dual-Probe Microfluidic Chip. Anal. Chem..

[13] Feng S., Shirani E., Inglis D.W. (2019). Droplets for Sampling and Transport of Chemical Signals in Biosensing: A Review. Biosensors.

[14] Zhu Y., Fang Q. (2013). Analytical detection techniques for droplet microfluidics—A review. Anal. Chim. Acta.

[15] Li R., Zhou Y., Chen Y., Zhou L., Yang Y., Xiao J., Liu G.L., Wang J., Huang L., Li Y. (2025). High-Electron-Mobility MXene-Enhanced Metasurface Biosensors Integrated with Microfluidics for Real-Time Multifunctional Monitoring. ACS Nano.

[16] Wang Y., Xue Y., Wang H., Qu Y., Zhang K., Shang L., Liang P., Chen F., Tang X., Luo W. (2025). Automated Laser-Assisted Single-Cell Sorting for Cell Functional and RNA Sequencing. ACS Sens..

[17] Gong P., Gao Z., Sun R., He B., Yuan F., Chen C., Zhao J., Su H., Wang L., Liu B. (2025). A microfluidic system for rapid enrichment and sensitive detection of *E. coli* based on bilayer membrane and high flux droplets. Talanta.

[18] Sun M., Fang Q. (2010). High-throughput sample introduction for droplet-based screening with an on-chip integrated sampling probe and slotted-vial array. Lab Chip.

[19] Zhang X., Sun R., Gong P., Al-Olayan E., Abukhadra M.R., Liu B., Su P., Zhang D., Feng S. (2025). A Large-field droplets for high-throughput *Escherichia coli* identification within one field of view. Talanta.

[20] Nan L., Lai M.Y.A., Tang M.Y.H., Chan Y.K., Poon L.L.M., Shum H.C. (2020). On-Demand Droplet Collection for Capturing Single Cells. Small.

[21] Song H., Ismagilov R.F. (2003). Millisecond kinetics on a microfluidic chip using nanoliters of reagents. J. Am. Chem. Soc..

[22] Wu M., Huang Y., Huang Y., Wang H., Li M., Zhou Y., Zhao H., Lan Y., Wu Z., Jia C. (2023). Droplet magnetic-controlled microfluidic chip integrated nucleic acid extraction and amplification for the detection of pathogens and tumor mutation sites. Anal. Chim. Acta.

[23] Chen Z., Yong Z., Leung C.W., Zhang X., Chen Y., Chan H.L., Wang Y. (2012). Time-variant 1D photonic crystals using flowing microdroplets. Opt. Express.

[24] Yadavali S., Jeong H.H., Lee D., Issadore D. (2018). Silicon and glass very large scale microfluidic droplet integration for terascale generation of polymer microparticles. Nat. Commun..

[25] Zhu P., Wang L. (2016). Passive and active droplet generation with microfluidics: A review. Lab Chip.

